# Supplementary material for: Target sample mining with modified activation residual network for speaker verification
Source: PLoS One. 2025 Apr 16;20(4):e0320256. doi: 10.1371/journal.pone.0320256 (PMC12002510; doi:10.1371/journal.pone.0320256)
Supplement: Attachment — Submitted filename: Comments for Author and Editors-D-24-37227.docx [file pone.0320256.s001.docx]

The study proposes a methodology to overcome some limitations in using traditional SoftMax methods. The writer proposed three methods and concluded that they enhance the accuracy of speaker verification tasks.

I think the study is trying to propose solutions to some limitations that can be very useful in the field. However, I think the writer should write more clearly to elevate the level of the paper and make it easier to follow. So, my recommendation is that the writers should edit the writing and add a discussion section then re-submit, if possible.

Here are some comments per section

**The Abstract**

It took the writer long to set the aim for the study. The abstract has to be concise and talks a bit about the background, the aim of the study, the methods used and the results of the study.

- The writers suggested a method to overcome some limitation with using SoftMax, but didn’t mention anything about the findings at the end of the abstract.
- Please avoid using ‘ novel’ when you propose a method/ an idea.
- I would avoid using ‘*has drawbacks*’ in the abstract, and instead use ‘some limitations’.
- When you talk about training, mention it is in machine learning. The abstract has to be understood without going back to other parts of the manuscript.
- The second sentence needs paraphrasing, it reads as if the discrepancy between predicted probability and the actual distribution is caused by the fact that SoftMax framework is suitable for multi-class classification problem in verification and training. Is that only SoftMax or are there joint functions that are used to make sure that there are no such discrepancies such as using loss function. Either way, please paraphrase for clarity.
- SoftMax instead of *softmax*
- As in the abstract we need to mention the methods used, it would be beneficial if you could explain in line or two what is the suggested method and what does it do?// what does it add to the ‘traditional methods’.
- You need to include the findings at the end of the abstract.
- The abstract and the conclusion should align, is it only one suggested method or three suggested methods?

**Introduction**

- The first part of the introduction reads as if the writer is listing the methods, some connection would help coherence.
- Please connect between paragraphs.
- Each paragraph should have a topic sentence that relates to the previous point, then all the sentences in the paragraph should explain link to other studies and you should critically say how is this point support your study follow PEEL & PEELC methods in writing paragraphs, please.
- In stead of saying ‘this section will…’ try to have some connection even with the use of subheadings.
- Add a Space between ‘.’ And the new sentences, please.
- Use references please when you talk about problems and the issues you raised.
- Avoid starting sentences with ‘and’ please.
- When introducing the data, just start talking about the data straight forward, please.
- You should have a subsection after talking about the data, which might be called ‘procedure’ what you did with the data. This helps the readers follow smoothly.

**Experimental analysis**

The first sentence here has to align with the objective of the study that you mentioned in the abstract and the conclusion. This has to be clarified in writing. Your proposed model is to use deep residual network and adaptive functions to improve speaker verification systems?, so make sure to emphasize that point.

This chapter would benefit from a discussion section where you present your results, explain them in relation to previous work in the field.

**Conclusion**

- This section starts by saying that there is a speaker verification method, but later on in the paragraph you say “*The experimental results indicate that all three proposed methods can enhance the accuracy of speaker verification tasks*”. You need to be precise and clear from the first sentence.
- Please paraphrase this sentence “*the stability and accuracy of deep networks in expressing speaker characteristics, and from the perspective of performance analysis in various aspects, the methods exhibit good performance*”
