# Supplementary material for: Target sample mining with modified activation residual network for speaker verification
Source: PLoS One. 2025 Apr 16;20(4):e0320256. doi: 10.1371/journal.pone.0320256 (PMC12002510; doi:10.1371/journal.pone.0320256)
Supplement: Attachment — Submitted filename: Comments 2.docx [file pone.0320256.s003.docx]

 Review Comments to the Author

Please use the space provided to explain your answers to the questions above. You may also include additional comments for the author, including concerns about dual publication, research ethics, or publication ethics. (Please upload your review as an attachment if it exceeds 20,000 characters)

Reviewer #1: This paper introduces a novel neural network architecture termed ResNet-P, which is constructed on the foundation of the Residual Network (ResNet) and incorporates a Parametric Rectified Linear Unit (PReLU) activation function. This modification is designed to enhance the network's capability to represent features and to mitigate issues related to gradient flow. Furthermore, the paper proposes an adaptive target function named SphereSpeaker, which is specifically tailored to optimize sample mining in speaker verification tasks. By incorporating strategies for balancing positive and negative samples and for mining challenging samples, the paper further refines the model's ability to handle imbalanced data during training, thereby improving the model's convergence rate and generalization capabilities. To diminish the overlap in similarity scores between positive and negative sample pairs, the paper introduces a method for adjusting similarities. Experiments conducted on the VoxCeleb1 database substantiate the efficacy of the proposed methods. However, the authors must address the following issues:

1. The paper exhibits subpar writing quality, with several sentences not adhering to standard academic expression. For instance, the phrase "This new network is named the Residual Network-PReLu, ResNet-P." should be rephrased for clarity and formality. Additionally, the use of "This chapter proposes..." in the Conclusion section is somewhat perplexing. The arbitrary naming of numerous acronyms also detracts from the manuscript's scholarly tone. It is recommended that the author revise the entire text for clarity, consistency, and adherence to academic writing standards.

Reply: Modifications have been made to the above question, all sentences containing "This chapter proposes..." have been modified.

2. The current manuscript appears to be a mere assemblage of technical elements without a coherent logical progression, which may impede the readers' comprehension. The author is advised to restructure the organization of the paper to enhance clarity and facilitate understanding.

Reply: Modified part of the paper structure, for example: after the data section, I added a subsection called "Procedure" that explains what I did with the data. This helps the reader's understanding.

3. The author's comparison of algorithms is outdated and does not reflect the current state of the art in speaker verification. It is recommended that the author updates the comparative analysis to include the most advanced algorithms in the field, such as transformer-based methods, to ensure the manuscript's relevance and contribution to the academic discourse.

Reply: In the Performance comparison and analysis section, three new comparison methods from the past two years have been added, and all three methods use the same dataset as this article, voxceleb1.

4. The author claims to have proposed a novel neural network architecture, namely ResNet-P (Residual Network-PReLu). However, it appears that numerous publications have already employed the combination of ResNet and PReLU, as referenced in [1-3]. Consequently, the innovative aspect of this manuscript seems insufficient. It is suggested that the author either provides a more substantial contribution or clearly delineates how their work differs from and improves upon the existing literature.
[1] Peng, S., Huang, H., Chen, W., Zhang, L., & Fang, W. (2020). More trainable inception-ResNet for face recognition. Neurocomputing, 411, 9-19.
[2] Trottier, L., Giguere, P., & Chaib-Draa, B. (2017, December). Parametric exponential linear unit for deep convolutional neural networks. In 2017 16th IEEE international conference on machine learning and applications (ICMLA) (pp. 207-214). IEEE.
[3] Zhao, M., Zhong, S., Fu, X., Tang, B., Dong, S., & Pecht, M. (2020). Deep residual networks with adaptively parametric rectifier linear units for fault diagnosis. IEEE transactions on industrial electronics, 68(3), 2587-2597.

Reply: The method of Resnet+PRelu is indeed applied in other deep learning fields. This paper considers applying the Resnet+PRelu network to the field of speaker verification. In the field of speaker verification, the application of this method is relatively rare. Through experimental comparison, it is found that using the Resnet+PRelu network has relatively good performance. At the same time, the article also made certain modifications to the description of the Resnet+PRelu method, deleting words such as "novel" and "new network". It has been changed to applying Resnet-P to the field of speaker verification.

5. The placement of the result figures at the end of the manuscript is not reader-friendly, as it disrupts the flow of information. Additionally, in Figure 3, the subfigures (e) and (f) are not aligned, which affects the visual presentation. It is recommended that the author reorganizes the manuscript to present the results in a more logical sequence as they are discussed in the text and ensures that all visual elements, such as figures, are properly aligned and formatted for clarity and aesthetic consistency.

Reply: Thank you for the guidance, the figures in the article have been organized to ensure good visual presentation.
